# Simultaneous Delivery of Econazole, Terbinafine and Amorolfine with Improved Cutaneous Bioavailability: A Novel Micelle-Based Antifungal “Tri-Therapy”

**DOI:** 10.3390/pharmaceutics14020271

**Published:** 2022-01-24

**Authors:** Si Gou, Michel Monod, Denis Salomon, Yogeshvar N. Kalia

**Affiliations:** 1 School of Pharmaceutical Sciences, University of Geneva, 1211 Geneva, Switzerland; Si.Gou@unige.ch; 2 Institute of Pharmaceutical Sciences of Western Switzerland, University of Geneva, 1211 Geneva, Switzerland; 3 Department of Dermatology, Laboratory of Mycology, Centre Hospitalier Universitaire Vaudois, 1011 Lausanne, Switzerland; Michel.Monod@chuv.ch; 4 Clinique Internationale de Dermatologie Geneve SA, 1201 Geneva, Switzerland; denis.salomon@cidge.ch

**Keywords:** antifungals, TPGS, micelles, cutaneous bioavailability

## Abstract

Lack of accurate diagnosis and the use of formulations designed to address the poor aqueous solubility of antifungal agents, but not optimized for delivery, contribute to unsatisfactory outcomes for topical treatment of cutaneous mycoses. The objective of this study was to develop a micelle-based antifungal formulation containing econazole (ECZ), terbinafine (TBF) and amorolfine (AMF) using D-α-tocopheryl polyethylene glycol succinate (TPGS) for simultaneous cutaneous delivery of three agents with complementary mechanisms of action. The antifungal “tri-therapy” micelle-based formulation containing 0.1% ECZ, 0.1% TBF and 0.025% AMF had a drug loading 10-fold lower than the “reference” marketed formulations (Pevaryl^®^, 1% ECZ; Lamisil^®^, 1% TBF; Loceryl^®^, 0.25% AMF). Finite dose application of the micelle-based formulation for 6 h resulted in a statistically equivalent deposition of ECZ (*p* > 0.05) and TBF (*p* > 0.05) from the 2 systems, and a 2-fold higher accumulation of AMF (*p* = 0.017). Antifungal concentrations above MIC_80_ against *Trichophyton rubrum* were achieved in each skin layer with the “tri-therapy”, which also exhibited a preferential deposition of each antifungal agent in pilosebaceous unit (PSU)-containing biopsies as compared with PSU-free biopsies (*p* < 0.05). A planned clinical study will test whether these promising results translate to improved therapeutic outcomes in vivo.

## 1. Introduction

Dermatomycosis is a widespread condition characterized by both superficial and subcutaneous infections of keratinous tissues [1,2]. Approximately 20% of the world’s population suffers from cutaneous mycoses; these are caused mostly by the anthropophilic dermatophyte *Trichophyton rubrum* [3,4,5], and the incidence has been increasing steadily, especially in patients with impaired immune functions [6].

Although standard treatments using azole compounds and terbinafine are effective in many cases of cutaneous dermatophytosis, oral medication for an extensive period of time may lead to significant side effects [7,8] and the treatment efficiency of antifungal drugs depends on their ability to achieve an effective concentration in the target skin layers. It is well recognized that topical administration of antifungal agents is desirable for the management of localized cutaneous mycoses due to the targeted delivery to the site of infection, which decreases the risk of side effects and drug interactions associated with systemic therapies [9].

However, the poor aqueous solubility of many antifungal agents makes their formulation difficult and results in limited cutaneous bioavailability. For example, econazole (ECZ, log P = 4.48 ± 0.53, aqueous solubility = 0.0057 mg/mL; pH = 7, 25 °C; log P values and aqueous solubility cited are predicted values calculated in SciFinder^®^ using Advanced Chemistry Development (ACD/Labs) Software V11.02 ©1994-2019 ACD/Labs) is the most prominent therapeutic agent employed topically to treat skin infections. However, it has been reported that ~90% of the ECZ present in the marketed formulation (Pevaryl^®^ cream; (1%, *w*/*w*)) remained on the skin surface following topical application, potentially leading to irritation, redness, burning or itching [10]. In addition, ECZ nitrate cannot be completely dissolved, leading to physical phase separation of the emulsion [11]. Excipients, e.g., emulsifiers and surfactants, used to solubilize ECZ and to improve formulation stability will lower its thermodynamic activity and thus reduce drug partitioning from the topical formulation to skin—rendering it more difficult to achieve therapeutic concentrations at the site of infection [10,12]. It has also been reported that the use of antimicrobial preservatives (e.g., benzoic acid) to prevent microbial contamination tends to generate an undesirable environment that can accelerate drug hydrolysis [13]. A number of novel strategies have been employed to address issues related to ECZ cutaneous bioavailability, including the development of polymeric micelles using methoxy-poly(ethylene glycol)-di-hexyl-substituted-poly(lactic acid (MPEG-hexPLA), which gave very promising results [14].

Despite appropriate formulation strategies, acquired resistance of dermatophytes, in particular, that of *Trichophyton indotineae* in India, is a serious emerging problem in several countries [15,16]. Therefore, combination therapy has been considered as an alternative means of achieving higher cure rates [17]. For example, the synergistic activity of amorolfine (AMF) with terbinafine (TBF) has been demonstrated in vitro and in animal studies [18]; combination of azoles with TBF or with AMF was also shown to be superior to monotherapy in clinical trials [19,20]. Considering the broad spectrum of activity and multiple enzyme targets possible by combining ECZ, TBF and AMF, it would be of great interest to develop a formulation for simultaneous topical cutaneous delivery of the three antifungal agents [21,22].

Due to the difference in their physicochemical properties—ECZ (log P = 4.48 ± 0.53, water solubility = 0.0057 mg/mL), TBF (log P = 5.58 ± 0.28, water solubility = 0.00093 mg/mL) and AMF (log P = 1.24 ± 0.28, water solubility = 9.2 mg/mL) [23]—it was decided to incorporate the highly lipophilic ECZ and TBF into polymeric micelles but separately, and then to combine the polymeric micelles containing ECZ or TBF with AMF solution. D-α-tocopheryl-polyethylene glycol 1000 succinate (TPGS), a water soluble, non-ionic derivative of natural vitamin E with polyethylene glycol 1000 (PEG), was selected as the copolymer to prepare the micelle formulations, since it is approved by the US Food and Drug Administration (FDA) and the European Medicine Agency (EMA) as a safe pharmaceutical ingredient [24].

To date, various TPGS-based drug delivery systems, including prodrugs [25], liposomes [26], micelles [27] and nanoparticles [28], have been developed for oral [29,30] and intravenous [31] administration. A recent publication reported a polymeric micelle gel formulation of adapalene (ADA) using TPGS for topical application; the polymeric micelle system increased drug solubility and enabled a selective, targeted drug delivery to the pilosebaceous unit (PSU) under finite dose conditions [32]. TPGS has also been employed for the development of sirolimus (SIR)-loaded polymeric micelles; the optimized formulation resulted in an increased SIR cutaneous bioavailability and a targeted epidermal delivery [33].

Here, we present the first report on the simultaneous cutaneous delivery of ECZ, TBF and AMF from a micelle-based formulation. The specific aims of the study were as follows: (i) to develop a triple antifungal micelle-based formulation containing ECZ, TBF and AMF using TPGS; (ii) to quantify the cutaneous delivery of each drug from the micelle-based formulation and to compare it to that from marketed formulations; (iii) to investigate the cutaneous biodistribution [34] and follicular delivery [35] of each drug from the triple antifungal micelle-based formulation.

## 2. Materials and Methods

### 2.1. Materials

TBF hydrochloride and AMF hydrochloride were purchased from Hangzhou Dayang Chem Co., Limited (Hangzhou, China). ECZ nitrate, TPGS (D-α-tocopheryl polyethylene glycol 1000 succinate), sodium chloride, isopentane, Dulbecco’s phosphate-buffered saline (DPBS; without calcium chloride and magnesium chloride) and Tween^®^ 80 were purchased from Sigma-Aldrich Chemie (Buchs, Switzerland). Acetone, methanol and acetonitrile (ACN) of analytical grade were purchased from Fisher Scientific (Reinach, Switzerland). Formic acid (extra pure 99%) was obtained from Biosolve Chemicals (Dieuze, France). OCT mounting medium was obtained from VWR Chemicals (Leuven, Belgium). All aqueous solutions were prepared using Milli-Q water (resistivity > 18 MΩ.cm; Zug, Switzerland). Marketed formulations of ECZ-Pevaryl^®^ Cream (1%, *w*/*w*), TBF-Lamisil^®^ Cream (1% *w*/*w*) and AMF-Loceryl^®^ Cream (0.25%, *w*/*w*) were purchased from a local pharmacy. The major ingredients of the formulations are: (i) Pevaryl^®^ Cream—macrogols (PEG-6 and PEG-32)/glycol stearate, oleoyl macrogolglycerides, liquid paraffin, butylhydroxyanisole (E320), benzoic acid (E210), flower perfume 4074, purified water; (ii) Lamisil^®^ Cream—sodium hydroxide, benzyl alcohol, sorbitan monostearate, cetyl palmitate, cetyl alcohol, stearyl alcohol, polysorbate 60, isopropyl myristate, demineralised water; (iii) Loceryl^®^ Cream—polyoxyl 40 stearate, stearyl alcohol, paraffin liquid, white soft paraffin, carbomer sodium hydroxide, disodium edetate, 2-phenoxyethanol.

### 2.2. Analytical Methods

#### 2.2.1. Quantification of ECZ, TBF and AMF by HPLC-UV

A high-performance liquid chromatography (HPLC) system consisting of an LPG-3400A pump equipped with a WPS-3000 autosampler, a thermostatted column compartment TCC-3x00, and a UVD-3000 detector (Dionex; Voisins LeBretonneux, France) was used to simultaneously quantify ECZ, TBF and AMF contents in the micelle formulation. Isocratic elution was performed using a Lichrospher^®^ RP-18, 5 µm, 125 × 40 mm column (BGB Analytik AG; Boeckten, Switzerland) that was maintained at 30 °C. The mobile phase consisted of a mixture of acetonitrile and water (70:30, *v*/*v*) containing 12 mM phosphoric acid and 12 mM trimethylamine. The flow rate and injection volume were 1.5 mL/min and 50 µL, respectively. ECZ, TBF and AMF were detected at 2.54 min, 4.18 min and 6.54 min at wavelengths of 214 nm, 224 nm and 219 nm, respectively; the total run time was 10 min. Chromeleon^®^ chromatography management software was used for integration and data analysis. The HPLC-UV method was validated according to ICH guidelines, the lower limits of detection (LOD) and lower limits of quantification (LOQ) were 1.0 and 5.0 µg/mL, respectively, for ECZ, TBF and AMF. Complete details are provided in the Supplementary Materials.

#### 2.2.2. Quantification of ECZ, TBF and AMF by UHPLC-MS/MS

An ultra-high performance liquid chromatography-MS/MS (UHPLC-MS/MS) method was developed and validated to evaluate the permeation and cutaneous biodistribution of ECZ, TBF and AMF. A Waters Acquity^®^ UPLC^®^ system (Baden-Dättwil, Switzerland) comprising a binary solvent pump, sample manager and Waters XEVO^®^ TQ-MS detector (Baden-Dättwil, Switzerland) was used for sample analysis. Chromatographic separation was performed using a XBridge^™^ BEH-C18 column (2.1 × 100 mm, 2.5 µm), and the column temperature was maintained at 40 °C. The mobile phase consisted of a mixture of acetonitrile and water (65:35, *v*/*v*) containing 0.1% formic acid, under isocratic conditions. Solvents were degassed in an ultrasonic bath before use (Branson Ultrasonics Corporation; Danbury, CT, USA). The flow rate was set at 0.4 mL/min and the injection volume was 5 µL. Nitrogen and argon were used as the drying gas and collision gas, respectively. The detection was performed by electrospray ionization in positive ion mode using multiple reaction monitoring, the mass transition ion pairs were *m*/*z* 381.07→125.01, 292.27→115.04 and 318.35→105.02 for ECZ, TBF and AMF, respectively. The cone voltage and collision energy were 38 V and 26 V, 28 V and 50 V, and 10 V and 38 V for ECZ, TBF, and AMF, respectively. Data processing was performed using MassLynx^®^ software. The method was validated according to ICH guidelines, the LOD and LOQ were 1.0 and 2.0 ng/mL for ECZ, TBF and AMF. Complete details are provided in the Supplementary Materials.

### 2.3. Development of TPGS Micelle-Based Antifungal “Tri-Therapy”

#### 2.3.1. Preparation of Single Drug-Loaded Micelle Formulation

ECZ- and TBF-loaded micelles were prepared separately using the solvent evaporation method. Briefly, a given amount of drug and copolymer were dissolved in 4 mL of acetone. The mixture was added dropwise under sonication (Branson Digital Sonifier^®^ S-450D; Carouge, Switzerland) to 8 mL of Milli-Q water. Acetone was evaporated at 40 °C using a rotary evaporator (Büchi RE 121 Rotavapor^®^; Flawil, Switzerland). The micelle solution was equilibrated under vacuum overnight and centrifuged at 10,000 rpm for 20 min to remove the free drug (Eppendorf Centrifugate 5804; Hamburg, Germany) and the supernatant was collected. TPGS copolymer concentration was fixed at 25 mg/mL, and micelles were prepared with a series of target drug loadings—50, 100, 200 and 400 mg DRUG/g COPOLYMER corresponding to drug:copolymer ratios of 1:20, 1:10, 1:5, 2:5, respectively.

#### 2.3.2. Characterization of Single Drug-Loaded Micelle Formulation

**Drug content**: ECZ and TBF contents in their respective micelle formulations were determined using the validated HPLC-UV method. The drug present in the intact micelles was released by diluting the formulation with acetonitrile and vortexing for 5 min. The drug content, drug loading and incorporation efficiencies were calculated using Equations (1)–(3), respectively, as follows:

(1) $$Drug\;content\;\left(mg\;drug/mL\;formulation\right)=\frac{mass\;of\;drug\;in\;formulation\;\left(\mathrm{mg}\right)}{volume\;of\;the\;formulation\;\left(\mathrm{mL}\right)}$$

(2) $$Drug\;loading\;\left(mg\;drug/g\;copolymer\right)=\frac{\left[drug\right]\;in\;the\;formulation\;\left(\mathrm{mg}/\mathrm{mL}\right)}{\left[copolymer\right]\;in\;the\;fromulation\;\left(g/\mathrm{mL}\right)}$$

(3) $$Incorporation\;efficiency\;\left(\%\right)=\frac{mass\;of\;drug\;incorporation\;into\;micelle\;\left(\mathrm{mg}\right)\;}{mass\;of\;drug\;introduced\;\left(\mathrm{mg}\right)}\times 100$$

**Size Characterization:** Micelle formulations were characterized for their hydrodynamic diameter (Z_av_), volume-weighted diameter (d_v_), number-weighted diameter (d_n_) and polydispersity index (P.I.) using dynamic light scattering (DLS) with a Zetasizer Nano-ZS (Malvern Instruments Ltd.; Malvern, UK). Measurements were performed in triplicate at an angle of 90° and at a temperature of 25 °C.

**Morphology:** Micelle morphology was studied by transmission electron microscopy (TEM) using an FEI Tecnai TM G2 Sphera (Eindhoven, The Netherlands) equipped with a high resolution 2000 × 2000 pixel digital camera. Samples were prepared using the negative staining method. Briefly, 5 µL of micelle solution was placed onto an ionized carbon-coated copper grid (0.3 Torr, 400 V for 20 s), which was then placed in contact with a 100 µL drop of saturated uranyl acetate aqueous solution for 1 s and then another drop of 100 µL for 30 s. The excess staining solution was removed, and the grid was dried under room temperature.

**Stability of micelle formulations:** Micelle solutions were stored at 4 °C for 4 weeks. To investigate the short-term stability, the amount of drug incorporated was quantified by the validated HPLC-UV method at predetermined time-points, on day 1, 7, 14, 21 and 28.

#### 2.3.3. Preparation of Triple Antifungal Drug-Loaded Micelle Formulation

ECZ- and TBF-loaded micelles showing at least 4 weeks’ stability at 4 °C were selected and combined with AMF solution by simply diluting the optimal formulations using ultra-pure water. Briefly, AMF was prepared at a concentration of 8.0 mg/mL, then 0.125 mL AMF solution was combined with ECZ-loaded micelles (2.60 mg/mL, 1.54 mL) and TBF-loaded micelles (2.37 mg/mL, 1.69 mL), and ultra-pure water was added to obtain a final volume of 4 mL. Drug content (0.1% ECZ, 0.1% TBF, 0.025% AMF) in the “tri-therapy” was confirmed by HPLC-UV analysis. The triple antifungal micelle-based formulation was characterized in terms of drug content, morphology and stability, as described above.

### 2.4. Skin Preparation

Fresh porcine ears were purchased from a local abattoir (CARRE; Rolle, Switzerland) shortly after sacrifice. After washing with cold running water, the porcine skin was carefully excised from the external region of the ear and was separated from the underlying cartilage using a scalpel and tweezers. Hair was removed from the skin surface using electric clippers. Then, the full-thickness skin (1.0–1.5 mm) was punched into round discs (diameter, 32 mm; area, 8 cm^2^) with a hydraulic press (Perkin-Elmer GmbH and Co. KG; Überlingen, Germany).

Human skin samples were collected immediately after surgery from the Department of Plastic, Aesthetic and Reconstructive Surgery, Geneva University Hospital (Geneva, Switzerland). The study was approved by the Central Committee for Ethics in Research (CER: 08-150 (NAC08-051); Geneva University Hospital). The hypodermis and fatty tissue were removed. The excised skin samples were punched out into 32 mm diameter circular discs (area, 8 cm^2^) and were subsequently horizontally sliced with a Thomas Stadie-Riggs slicer (Thomas Scientific; Swedesboro, NJ, USA) to a thickness of ∼1000 µm.

The processed skin samples were stored at −20 °C for a maximum period of 2 months. Frozen skin samples (porcine or human) were equilibrated in NaCl solution (0.9%, *w*/*w*) for 30 min before permeation experiment.

### 2.5. Evaluation of Cutaneous Delivery In Vitro

Skin transport studies were performed in vitro using standard Franz vertical diffusion cells (Millan SA; Meyrin, Switzerland) with a formulation contact area of 2 cm^2^. Porcine or human skin samples were mounted in the cells and equilibrated with phosphate-buffered saline (PBS, pH = 7.4) for 30 min. The micelle-based antifungal “tri-therapy” formulation (50 µL/cm^2^ for infinite dose conditions and 10 µL/cm^2^ for finite dose conditions) or marketed formulation (again either 50 mg/cm^2^ or 10 mg/cm^2^ for infinite and finite dose conditions) was applied to the exterior surface of porcine or human skin for a given application time (1, 6 or 12 h). The receptor compartments were filled with 10 mL PBS containing Tween 80 (0.1%, *w*/*w*) to maintain sink conditions. The receptor compartment was stirred at 250 rpm and maintained at 37 °C throughout the experiment. Aliquots (1 mL) of the receptor phase were withdrawn upon completion of the skin delivery experiment to quantify the amount of drug permeated across the skin using the validated UHPLC-MS/MS method. The diffusion cells were then dismantled, and each skin sample was carefully washed with PBS containing 1% Tween 80 to ensure any residual formulation was completely removed from the surface. Then, the skin samples were dried with a cotton swab and cut into small pieces; drug deposited in the skin was extracted by soaking the pieces in a mixture of 20 mL ACN:H_2_O (90: 10, *v*/*v*) overnight under constant stirring at room temperature. Then, 1.5 mL samples were withdrawn, centrifuged at 10,000 rpm for 20 min and filtered through 0.22 μm nylon membrane filters. The filtrates were then analyzed using the validated UHPLC-MS/MS method. Complete details are provided in the Supplementary Materials.

### 2.6. Investigation of Antifungal Biodistribution Profile

Cutaneous biodistribution of each antifungal drug from the micelle-based antifungal “tri-therapy” was investigated. The micelle-based antifungal “tri-therapy” formulation was applied under finite dose conditions (10 µL/cm^2^) for 6 h. At the end of the skin delivery experiment, a 0.5 cm^2^ disc was punched out from the central area of the skin samples. The small skin disc was snap-frozen in isopentane cooled with liquid nitrogen and mounted on a cryotome (Microm HM 560 Cryostat; Walldorf, Germany) to obtain horizontal (XY plane) lamellae with a thickness of 40 µm. Each lamella was collected in a 2 mL Eppendorf tube; all lamellae and the remaining piece of skin were extracted in 1.5 mL of ACN:H_2_O (90:10) overnight with continuous stirring at room temperature. The samples were centrifuged at 10,000 rpm for 20 min and the concentration of ECZ, TBF and AMF was quantified by the validated UHPLC-MS/MS method. A total of 20 lamellae obtained from each skin sample (from the skin surface to a depth of ~800 µm), provided the biodistribution profile of the 3 antifungal drugs from the stratum corneum, through the viable epidermis and to the upper dermis.

### 2.7. Antifungal Delivery to the PSU

After application of the micelle-based antifungal “tri-therapy” formulation was completed under finite dose conditions (10 µL/cm^2^) for 6 h, PSU-containing and PSU-free biopsies were punched out using a previously validated punch biopsy method to evaluate the ability of micelles to target the delivery of the antifungal agents to follicular structures [35]. Briefly, the skin was cleaned after the completion of delivery experiments using the protocol described above. The PSU-containing biopsy and PSU-free biopsy were harvested using a 1 mm diameter punch (Berg & Schmid HK 500; Urdorf, Switzerland). The harvested biopsy samples were inspected using a Leica S6D microscope (Leica Microsystems (Schweiz) AG; Heerbrugg, Switzerland) to confirm the presence of an entire PSU or to establish that the skin sample was a PSU-free region. Antifungal agents deposited in each biopsy sample were extracted overnight using 100 µL of ACN:H_2_O (90:10, *v*/*v*) at room temperature on a shaker bath at 150 rpm in an Eppendorf tube. The samples were centrifuged at 10,000 rpm for 15 min; ECZ, TBF and AMF contents were quantified using UHPLC-MS/MS.

### 2.8. Data Analysis

Data were expressed as mean ± standard deviation (mean ± SD). Outliers determined using the Dixon test were discarded. Statistical differences were determined by a one-way ANOVA followed by a Student–Newman–Keuls test (*p < 0.05*), which were performed using Sigma Plot 12.5 (Systat Software Inc., San Jose, CA, USA).

## 3. Results and Discussion

### 3.1. Characterization and Properties of Single Antifungal Drug-Loaded Micelles

**Drug content:** A series of ECZ- and TBF-containing micelles were prepared to investigate the effect of the drug:polymer ratio (1:20, 1:10, 1:5, 2:5) on drug incorporation (Table 1. **E1–E4** for ECZ and **T1–T4** for TBF). Micelles with incorporation efficiencies (I.E.) ranging from 36.93 ± 0.42 to 109.72 ± 0.64% for ECZ and 92.53 ± 0.58 to 107.57 ± 3.28% for TBF were obtained, corresponding to drug contents (D.C.) from 1.40 ± 0.01 to 5.19 ± 0.02 mg/mL for ECZ and from 1.32 ± 0.02 to 9.50 ± 0.43 mg/mL for TBF. The solubility of ECZ and TBF were significantly increased—from 0.0057 to 5.19 ± 0.02 mg/mL (910-fold) and 0.00093 to 9.50 ± 0.43 mg/mL (~10,000-fold), respectively. The drug loading (D.L.) for ECZ peaked at 200 mg DRUG/g POLYMER (**E3** with a D:P ratio of 1:5) before decreasing sharply; however, for TBF, it continued to increase and a D.L. of 380.00 ± 17.24 mg DRUG/g COPOLYMER was achieved for a theoretical loading of 400 mg DRUG/g COPOLYMER. This suggested that TBF had superior affinity for TPGS compared with ECZ.

**Size characterization:** ECZ- and TBF-loaded micelles were characterized using dynamic light scattering (DLS) (Table 2). All formulations presented homogeneous nanometer scale size, which increased with increasing drug loading (D.L.). The values obtained for **E3** were a little higher; the statistically significant difference could have been due to the greater drug content and the formation of aggregates. ECZ micelles (**E1** and **E2**) were of a comparatively smaller size than those containing TBF (**T1, T2, T3** and **T4**) with hydrodynamic diameters (Z_av_) from 4.675 to 4.982 nm vs. 7.084 to 9.116 nm, but in all cases < 10 nm. The results were in accordance with number weighted diameters (d_n_) and volume weighted diameter (d_v_), which were 3.1–3.3 nm and 3.8–3.9 nm for ECZ, and 5.2–6.7 nm and 6.1–7.9 nm for TBF, respectively. It was reported that micelle size is influenced by structural parameters of the polymer (such as composition, length and molecular mass) polymer concentration, drug loading and drug polymer interaction. It was observed that at a given TPGS polymer concentration (25 mg/mL), a greater amount of TBF was solublized in the system, implying there are more drug molecules entrapped in each individual micelle architecture, which is consistent with the larger diameters of the TBF micelles.

**Figure 1:**
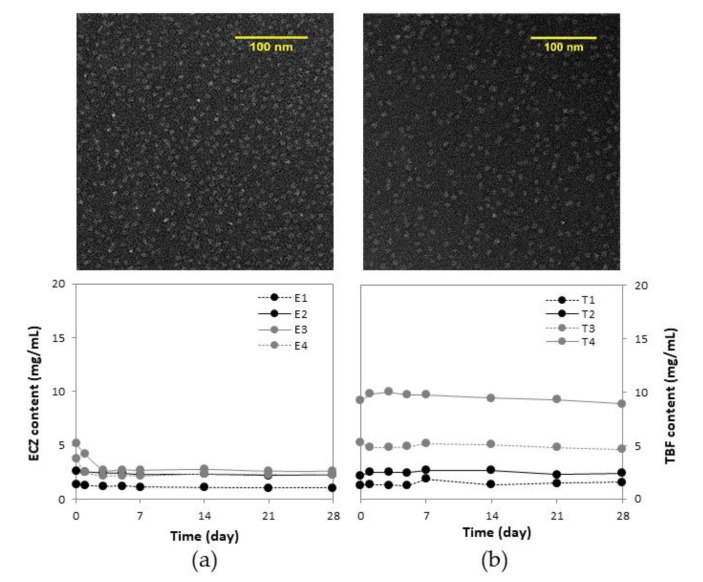
Characterization of (**a**) ECZ-loaded and (**b**) TBF-loaded micelles with respect to morphology using TEM (magnification 62,000×) and stability at 4 °C, over 4 weeks (*n* = 3).

**Morphology:** TEM images of ECZ-loaded (Figure 1a) and TBF-loaded (Figure 1b) micelles provided visual evidence that the micelles (white) were spherical in shape with diameters in the nanometer range on a black background of the ionized carbon-coated copper grid that was used for sample preparation. Observation of the micelle formulations using TEM was in a good agreement with the particle size analysis by DLS.

**Formulation stability:** With a polymer concentration of 25 mg/mL, **E3** achieved the highest ECZ concentration (Figure 1a). However, a sharp decrease in drug content was observed within the first 3 days (from 5.19 ± 0.02 to 2.73 ± 0.01 mg/mL) and after 28 days, the concentration was 2.61 ± 0.20 mg/mL. The trend was corroborated by visual observation of the formation of ECZ crystals at the bottom of the vial. This suggested the batch with the highest drug loading was transiently supersaturated and ECZ precipitated out from solution during storage [36]. Therefore, **E2**, with an ECZ content of 2.60 ± 0.01 mg/mL and an excellent stability after storage for one month was considered as the optimal formulation.

In the case of TBF (Figure 1b), drug content went up to 9.50 ± 0.43 mg/mL (**T4**) and all formulations were stable after one month. Recent studies have shown that the incorporation efficiency and stability of the micelle formulation were determined by drug–copolymer affinity, which was achieved via more complex molecular mechanisms of interaction than simple hydrophobic interactions [37]. According to an investigation into the incorporation of 18 different drugs in MPEG-hexPLA micelles, drug–polymer compatibility was influenced in decreasing order by the number of hydrogen bond donors (H_d_), log *p*, number of hydrogen acceptors (H_a_) and the aqueous solubility [38]. Both ECZ and TBF contain one H_d_ group, the higher log *p* value of TBF may explain the higher drug loading and better stability. However, in order to have the same polymer:drug ratio as that in the optimal ECZ formulation, **E2,** and thereby avoid any effects due to differences in polymer:drug ratio, **T2** was considered as the optimal TBF formulation, corresponding to a 456- and 2548-fold enhancement of aqueous solubility for ECZ (**E2** from 0.0057 to 2.60 ± 0.01 mg/mL) and TBF (**T2** from 0.00093 to 2.37 ± 0.01 mg/mL), respectively.

### 3.2. Triple Antifungal Drug-Loaded Micelle Formulation

To carry out comparisons with the marketed formulations, Pevaryl^®^ Cream (1% ECZ), Lamisil^®^ Cream (1% TBF), and Loceryl^®^ Cream (0.25% AMF), it was decided to prepare an antifungal “tri-therapy” formulation, but with 10-fold lower drug concentrations in the antifungal drug-loaded micelles.

**Formulation B** (0.1% ECZ, 0.1% TBF and 0.025% AMF) was obtained by simply diluting **Formulation A**—the optimal ECZ micelles (**E2**) and TBF micelles (**T2**)—using ultra-pure water and combing with AMF solution (Table 3).

**Figure 2:**
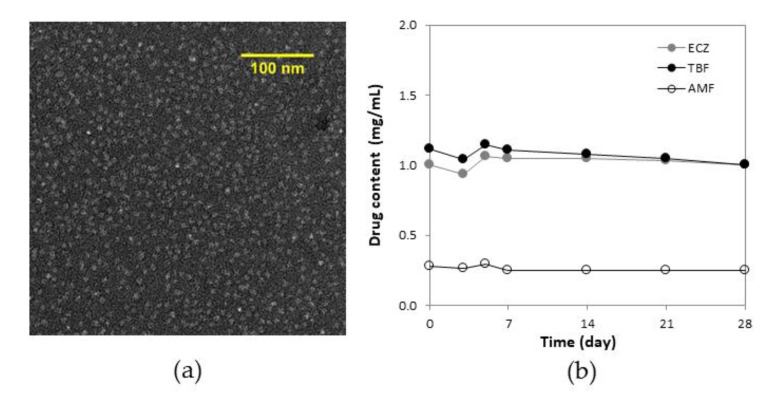
Characterization of the antifungal “tri-therapy”: (**a**) morphology under TEM (magnification 62,000×); (**b**) formulation stability by monitoring drug content as a function of time during storage for 4 weeks at 4 °C (*n* = 3).

The micelle-based formulation containing the three antifungal drug was studied using TEM and the images confirmed that micelle structure and morphology were not affected by dilution, and that the spherical shape and nanometer size were retained (Figure 2a). Drug content measured in the supernatant was stable during storage for one month, suggesting that the entrapped ECZ and TBF were localized in the micelle and the introduction of AMF in the aqueous solution did not influence formulation stability (Figure 2b).

### 3.3. Cutaneous Delivery of the Antifungal Drugs

#### 3.3.1. Antifungal Delivery under Infinite Dose

**Figure 3:**
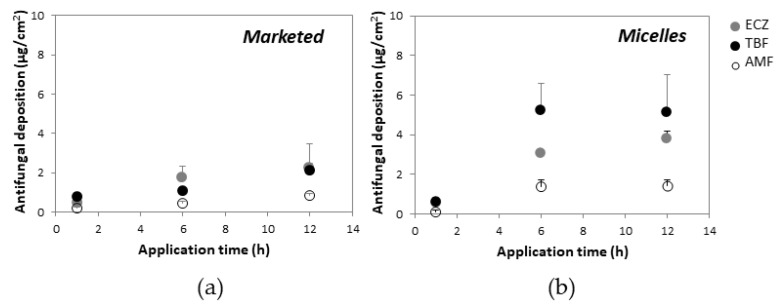
Antifungal deposition in porcine skin under infinite dose from (**a**) marketed formulations and (**b**) micelle-based formulation as a function of application time (mean ± SD, *n* ≥ 5), permeation was not detected (below LOQ (2.0 ng/mL)) after 12 h.

The triple antifungal micelle-based formulation and marketed formulation were applied for three different application times (1, 6 and 12 h) in order to evaluate antifungal delivery kinetics into and through porcine skin (Figure 3). These first experiments were performed under infinite dose conditions (50 µL/cm^2^—i.e., 0.05 mg ECZ and TBF, 0. 0125 mg AMF per cm^2^ for the triple antifungal micelle-based formulation; 50 mg/cm^2^—i.e., 0.5 mg ECZ and TBF, 0.125 mg AMF per cm^2^ for the marketed products). The amounts of each antifungal permeated across porcine skin, even after the longest application time from either the triple antifungal micelle-based formulation or the marketed formulations, were below the LOQ (2.0 ng/mL) of the UHPLC-MS/MS method. It should be noted that the micelle-based formulation produced a higher skin deposition than that from the marketed formulations at each time point (Figure 3), which might be attributed in part to the smaller size of the carrier yielding a higher contact surface with skin (i.e., ECZ micelles have diameters (Z_av_) of ~4 nm vs. Pevaryl^®^ liposomes that are reported to have diameters (Z_av_) of 160–200 nm [39]). The micelle structure will most likely collapse upon contact with the skin surface and release ECZ and TBF into the aqueous environment forming a supersaturated solution in situ given their poor aqueous solubility. The higher thermodynamic activities in the supersaturated system would facilitate drug partitioning into the stratum corneum. However, the drugs might also have a tendency to crash out of the solution, forming crystals in contact with the rough surface of the stratum corneum along with water evaporation in the donor compartment, which would obviously prevent transport [40].

Increasing the application time from 1 h to 6 h resulted in a significant increase in antifungal deposition from the micelle-based solution and the marketed creams, but the amounts accumulated from micelle solution after 12 h were statistically equivalent to those obtained at 6 h. It was surprising that AMF—existing as a free drug in the micelle solution—also achieved a higher skin deposition in comparison with the marketed formulation. It was hypothesized that (i) the hydrated environment of the aqueous solution facilitated its penetration, and (ii) TPGS might function as a penetration enhancer promoting diffusion by altering the skin structure [41].

**Figure 4:**
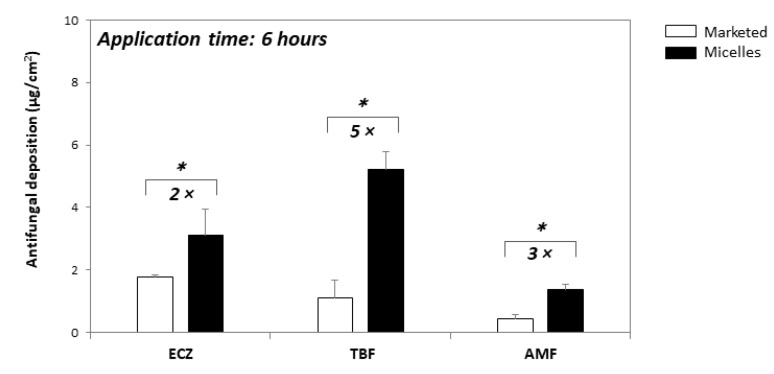
Comparison of the amount of each antifungal deposited from marketed formulations and from the “tri-therapy” following formulation application under infinite dose for 6 h (mean ± SD, *n* ≥ 5). * *p* < 0.05.

Considering the recommended therapeutic regimen (application 2–3 times per day) and the fact that the formulations might be washed off with showering, it was decided to focus on the experiments using an application time of 6 h—cutaneous delivery of ECZ, TBF and AMF from the “tri-therapy” micelle-based formulation under infinite dose was superior to that from Pevaryl^®^ (3.09 ± 1.06 vs. 1.76 ± 0.59 µg/cm^2^), Lamisil^®^ (5.23 ± 1.35 vs. 1.09 ± 0.09 µg/cm^2^), and Loceryl^®^ (1.37 ± 0.34 vs. 0.43 ± 0.14 µg/cm^2^), respectively (Figure 4); thus demonstrating that the triple antifungal micelle-based formulation enabled simultaneous delivery of ECZ, TBF and AMF into skin and, moreover, resulted in a 2- to 5-fold higher deposition with 10-fold lower drug loading as compared with the marketed formulations.

#### 3.3.2. Antifungal Delivery under Finite Dose

**Figure 5:**
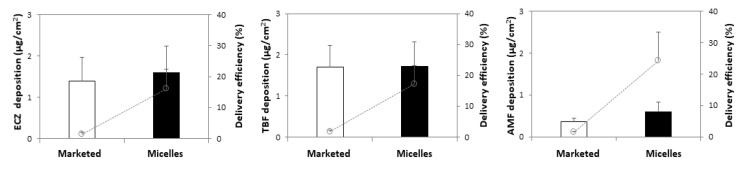
Comparison of ECZ, TBF and AMF delivery to the skin (left, middle and right panels) from marketed formulations and from the “tri-therapy” following formulation application under finite dose for 6 h (mean ± SD, *n* ≥ 5).

Finite dose (10 mg/cm^2^ or 10 µL/cm^2^) conditions have been suggested by the OECD guideline for in vitro skin absorption studies to mimic better the actual conditions of use and to decrease the effect of excipients. Therefore, 20 mg of each marketed formulation or 20 µL micelle formulation was applied on the skin (per 2 cm^2^)—corresponding to 0.2 mg ECZ, 0.2 mg TBF and 0.05 mg AMF in marketed formulations (and 0.1, 0.1 and 0.025 mg/cm^2^, respectively); 0.02 mg ECZ, 0.02 mg TBF and 0.005 mg AMF in micelle-based formulation (0.01, 0.01 and 0.0025 mg/cm^2^, respectively). Antifungals incorporated in the micelles—ECZ and TBF—achieved a skin deposition of 1.61 ± 0.62 and 1.71 ± 0.60 µg/cm^2^, respectively, following finite dose application for 6 h, which was statistically equivalent to the amount delivered from the marketed formulations—1.39 ± 0.58 and 1.70 ± 0.53 µg/cm^2^ (*p* > 0.05)—despite the 10-fold lower loading, and AMF resulted in a 2-fold higher accumulation from micelle solution as compared with the marketed cream (0.60 ± 0.23 vs. 0.36 ± 0.09 µg/cm^2^) (Figure 5). Thus, the finite dose condition had less impact on AMF than ECZ and TBF delivery from the micelle solution. Despite the similar absolute amounts observed in the antifungal deposition, it should be noted that the micelle carrier systems yielded significantly increased delivery efficiency for each drug—from 1.39 ± 0.58, 1.70 ± 0.53, and 1.43 ± 0.34% to 16.11 ± 6.19, 17.12 ± 6.01, and 24.12 ± 9.15% for ECZ, TBF, and AMF, respectively (Figure 5).

In vitro studies performed under finite dose provide a better simulation of in vivo application. In the case of the marketed formulations, there were no statistically significant differences (*p* > 0.05) in skin deposition upon passing from infinite to finite dose conditions. However, 2–3-fold reductions in antifungal deposition in skin were observed for the micelle-based formulation. It could be explained by water evaporation and subsequent crystallization of drug molecules and/or depletion from the micelle solution under finite dose conditions, which would clearly hinder partitioning and penetration of the antifungal drugs into the stratum corneum. However, the decrease in skin deposition was less than the 5-fold change in the amount of formulation applied. The impact of finite dose on nanocarrier formulation needs to be further elucidated and methods devised to offset the effect of water evaporation.

**Figure 6:**
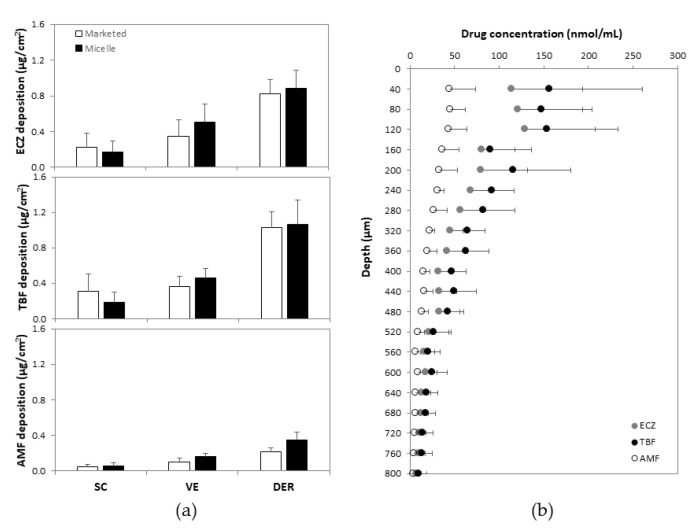
(**a**) Comparison of ECZ, TBF and AMF deposited in different anatomical regions in the skin—SC (stratum corneum, 0–40 µm), VE (viable epidermis, 40–160 µm) and DER (dermis, 160 µm)—from marketed formulations and from the “tri-therapy” (mean ± SD, *n* ≥ 5); (**b**) cutaneous biodistribution profile of ECZ, TBF and AMF from the “tri-therapy” (mean ± SD, *n* ≥ 5).

Although the results confirmed that the triple antifungal micelle-based formulation was able to deliver ECZ, TBF and AMF simultaneously into skin, it is important to know the spatial distribution of the drug molecules and to identify to which skin layer they had been delivered. Therefore, the porcine skin was cryotomed into a series of lamellae, each with a thickness of ~40 µm, following the permeation experiment and samples were analyzed using the validated UHPLC-MS/MS methods. Antifungal deposition in each section of porcine skin was quantified, the amounts of ECZ (0.57 ± 0.25 vs. 0.68 ± 0.23 µg/cm^2^), TBF (0.67 ± 0.22 vs. 0.64 ± 0.16 µg/cm^2^) and AMF (0.14 ± 0.06 vs. 0.21 ± 0.06 µg/cm^2^) deposited in epidermis (0–160 µm, stratum corneum and viable epidermis) from marketed and micelle-based formulations were statistically equivalent (Figure 6a), indicating that the same amount of antifungal was delivered (keeping in mind the 10-fold lower drug loading in the “tri-therapy”). The highest drug concentration was detected in the first skin layer sample—stratum corneum (0–40 µm)—and the concentrations decreased with increasing skin depth; thus, a concentration gradient was observed within the skin tissue (Figure 6b). However, the concentration of each antifungal agent in the epidermis—the therapeutic compartment—was significantly higher than the MIC_80_ (concentration of antifungal producing a growth inhibition of 80% or more) against the main dermatophytic pathogen, *Trichophyton rubrum* (MIC_80_ = 0.02, 1.31 and 0.08 nmol/mL for ECZ, TBF and AMF, respectively) [21]. Complete details are provided in the Supplementary Materials.

In future studies, desorption electrospray ionization-mass spectrometry imaging (DESI-MSI) will be employed to visualize the spatial distribution of these molecules in the skin and thereby identify penetration pathways—this should also serve to highlight the potential contribution of appendageal routes to drug delivery. We have recently described how this technique was used to visualize the molecular distribution of ECZ and TPGS in 40 µm thick horizontal sections and vertical cross-sections of the skin following topical micelle application; the co-localization of drug and excipient with endogenous skin components provided insight into the respective cutaneous penetration pathways [42].

### 3.4. Targeted Delivery to the PSU

**Figure 7:**
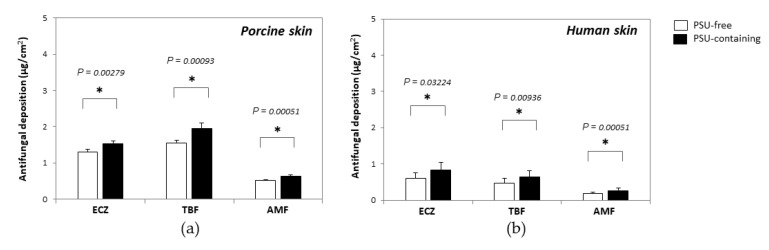
Comparison of antifungal deposition in PSU-free and PSU-containing biopsies: (**a**) porcine ear skin and (**b**) human abdominal skin (mean ± SD, *n* ≥ 5). Statistically significant differences are indicate by the *p*-value in each figure.

Based on the fact that the spores invade skin tissue by secreting germ tubes, and that the pathogen tends to accumulate in the hair follicle structure during infection, selective delivery of the antifungal agents to the PSU might be expected to produce a better therapeutic effect. Therefore, PSU-containing biopsies or PSU-free biopsies were punched out upon completion of the skin delivery experiment, the integrity of their structure was visually confirmed under light microscopy and drug amounts quantified (Figure 7). In porcine skin samples (Figure 7a), slightly greater amounts of ECZ and TBF were deposited in the PSU-containing biopsies (1.52 ± 0.09 and 1.94 ± 0.16 µg/cm^2^, *p* = 0.0027) than in PSU-free biopsies (1.30 ± 0.08 and 1.54 ± 0.08 µg/cm^2^, *p* = 0.0009) after application of micelle formulation under finite dose for 6 h. AMF deposition in PSU-containing biopsies was also higher than that in PSU-free biopsies (0.64 ± 0.04 vs. 0.52 ± 0.02 µg/cm^2^, *p* = 0.0005), which was in accordance with the reports on caffeine (log P = −0.07) transport through hair follicles in vitro [43] and in vivo [44]; this points to a more important role of the follicular pathway in the permeation of hydrophilic molecules.

Following finite dose application to human skin for 6 h, a statistically greater deposition of ECZ, TBF and AMF was also observed in the PSU-containing biopsies (1.61 ± 0.62, 1.71 ± 0.60, 0.60 ± 0.23 µg/cm^2^, respectively) than in PSU-free biopsies (0.82 ± 0.16, 1.11 ± 0.19, 0.31 ± 0.05 µg/cm^2^, respectively) (Figure 7b). Indeed, the selectivity was more pronounced in the case of human skin: 1.96-, 1.54- and 1.94-fold greater amounts of ECZ, TBF and AMF being measured in the PSU-containing biopsies, respectively. These results further confirmed the preferential delivery of nanocarriers (and AMF, a hydrophilic molecule) into the PSU [32,35]. It was noted that the selectivity of the “tri-therapy” was less pronounced compared with the results for adapalene (4.5- and 3.3-fold for micelle solution and micelle gel, respectively) [32] and spironolactone (5-fold) [45]. The hypothesis was that the available volume within the pilosebaceous unit limited the number of nanocarriers that could be accumulated in each structure. When the “tri-therapy” containing three molecules was applied on the skin surface, TBF- and ECZ-loaded micelles were competing to reside in the PSU, while the co-formulation had no influence on their distrubution in PSU-free skin. Therefore, the “tri-therapy” yielded a lass marked selectivity for deposition in the PSU for each drug molecule.

## 4. Conclusions

An antifungal “tri-therapy” containing 0.1% ECZ, 0.1% TBF and 0.025% AMF was successfully developed using D-α-tocopheryl polyethylene glycol succinate (TPGS) and enabled simultaneous delivery of the three antifungal agents. The novel polymeric micelle carrier system yielded a statistically equivalent deposition of ECZ, TBF and AMF in the treatment compartment, the epidermis, as compared to the marketed formulations under finite dose condition, despite a 10-fold lower drug content. Drug concentrations of each antifungal agent in all the skin layers were significantly higher than their MIC_80_ against *Trichophyton rubrum*. A preferential deposition of each antifungal agent in PSU-containing biopsies was observed in porcine, and more importantly, human skin, and a higher therapeutic efficacy might be expected from the synergistic effect of the antifungal combination that target key enzymes involved in ergosterol biosynthesis. In conclusion, the study employed a novel nanocarrier-based formulation to enable polypharmacy through simultaneous delivery of drugs with complementary mechanisms of action and used advanced experimental methods to investigate the cutaneous biodistribution of the three drugs and the ability of the formulation to target appendageal structures. The next steps in the project will involve the following: (i) a preliminary investigation in vitro into the efficacy of this novel formulation against fungal strains; (ii) computational studies to simulate the process of self-assembly and drug loading and distribution in the micelle. These will be extremely useful, together with further experimental data, in elucidating the molecular interactions involved in the formation of drug-loaded polymeric micelles and to predict the compatibility of a specific drug with a given copolymer.

## Figures and Tables

**Table 1:** Drug content of ECZ- and TBF-loaded micelles.

# *^a^*	P.C.	D:P ratio	T.L.	ECZ			TBF		
				D.L.	I.E.	D.C.	D.L.	I.E.	D.C.
1	25	1:20	50	55.96 ± 0.33	109.72 ± 0.64	1.40 ± 0.01	52.78 ± 0.85	103.48 ± 1.67	1.32 ± 0.02
2	25	1:10	100	104.18 ± 0.42	103.14 ± 0.42	2.60 ± 0.01	94.85 ± 0.59	92.53 ± 0.58	2.37 ± 0.01
3	25	1:5	200	207.74 ± 0.92	102.33 ± 0.45	5.19 ± 0.02	216.21 ± 6.58	107.57 ± 3.28	5.41 ± 0.16
4	25	2:5	400	151.43 ± 1.71	36.93 ± 0.42	3.79 ± 0.04	380.00 ±17.24	94.76 ± 4.30	9.50 ± 0.43

*^a^* Formulations **# 1–4** (ECZ: **E1–E4** and TBF: **T1–T4**); polymer content (P.C., mg/mL); drug:polymer ratio (D:P ratio); target loading (T.L., mg DRUG/g COPOLYMER); drug loading (D.L., mg DRUG/g COPOLYMER); incorporation efficiency (I.E., %); drug content (D.C., mg/mL).

**Table 2:** Size characterization of ECZ- and TBF-loaded micelles.

#*^a^*	ECZ				TBF			
	Z_av_	P.I.	d_n_	d_v_	Z_av_	P.I.	d_n_	d_v_
1	4.982	0.184	3.315	3.908	7.084	0.051	5.257	6.162
2	4.675	0.122	3.148	3.862	7.114	0.057	5.274	6.189
3	8.105	0.061	5.927	7.018	8.561	0.195	6.204	7.319
4	5.118	0.145	3.175	4.019	9.116	0.062	6.738	7.937

*^a^* Formulations # **1**–**4** (ECZ: **E1–E4** and TBF: **T1–T4**); hydrodynamic diameter (Z_av_, nm); polydispersity index (P.I.); number-weighted diameter (d_n_, nm); volume-weighted diameter (d_v_, nm).

**Table 3:** Content of ECZ- and TBF-loaded micelles.

F	ECZ				TBF			
	D.L.	I.E.	D.C.	P.C.	D.L.	I.E.	D.C.	P.C.
**A**	104.18 ± 0.42	103.14 ± 0.42	2.60 ± 0.01	25	94.85 ± 0.59	92.53 ± 0.58	2.37 ± 0.01	25
**B**	104.18 ± 0.42	103.14 ± 0.42	1.01 ± 0.01	9.6	94.85 ± 0.59	92.53 ± 0.58	1.08 ± 0.02	10.5

Formulation (F; B was obtained by a dilution of A-the optimal ECZ- and TBF-loaded formulation—E2, T2); drug loading (D.L., mg DRUG/g COPOLYMER); incorporation efficiency (I.E., %); drug content (D.C., mg/mL); copolymer content (P.C., mg/mL).

## Data Availability

All data available are reported in the article.

## Supplementary Materials

The following are available online at https://www.mdpi.com/article/10.3390/pharmaceutics14020271/s1, Figure S1: Chromatograms of (a) blank solvent, (b) a mixture of ECZ (50 µg/mL), TBF (50 µg/mL) and AMF (50 µg/mL) standard, Figure S2: MRM monitoring for ECZ (381.07→125.01 transition), TBF (292.27→115.04 transition) and AMF (318.35→105.02 transition) in skin sample. Chromatograms of blank solvent, skin matrix and a mixture of ECZ (1 ng/mL), TBF (1 ng/mL) and AMF (1 ng/mL) standard, Table S1: Intra- and inter-day accuracy and precision for simultaneous quantification of ECZ, TBF and AMF in solvent (values are given as mean ± SD), Table S2: MS/MS settings for detection of ECZ, TBF or AMF, Table S3: Intra- and inter-day accuracy and precision for simultaneous quantification of ECZ, TBF and AMF in skin samples (values are given as mean ± SD), Table S4: Antifungal solubility test, Table S5: Recovery of ECZ, TBF and AMF from skin extraction (values are given as mean ± SD), Table S6: In vitro antifungal activity of ECZ, TBF and AMF against dermatophyte, molds and yeasts.

## Abbreviations

ACN—acetonitrile; ADA—adapalene; AMF—amorolfine; D.C.—drug contents; D.L.—drug loading; DLS—dynamic light scattering; d_n_—number-weighted diameter; DPBS—Dulbecco’s phosphate-buffered saline; D:P ratio—drug:polymer ratio; d_v_—volume-weighted diameter; ECZ—econazole; EMA—European Medicine Agency; FDA—Food and Drug Administration; H_a_—hydrogen acceptors; H_d_—hydrogen bond donors; HPLC—high-performance liquid chromatography; I.E.—incorporation efficiencies; LOD—lower limits of detection; LOQ—lower limits of quantification; MPEG-hexPLA—methoxy-poly(ethylene glycol)-di-hexyl-substituted-poly(lactic acid); PEG—polyethylene glycol; P.I.—polydispersity index; PSU—pilosebaceous unit; SD—standard deviation; SIR—sirolimus; TEM—transmission electron microscopy; TBF—terbinafine; T.L.—target loading; TPGS—D-α-tocopheryl polyethylene glycol succinate; UHPLC-MS/MS—ultra-high performance liquid chromatography-MS/MS; Z_av_—hydrodynamic diameter.
