# Supplementary material for: Simultaneous Delivery of Econazole, Terbinafine and Amorolfine with Improved Cutaneous Bioavailability: A Novel Micelle-Based Antifungal “Tri-Therapy”
Source: Pharmaceutics. 2022 Jan 24;14(2):271. doi: 10.3390/pharmaceutics14020271 (PMC8880516; doi:10.3390/pharmaceutics14020271)
Supplement: Supplementary file 1 [file pharmaceutics-14-00271-s001.zip › pharmaceutics-1555217-supplementary.pdf]

pharmaceutics

MDPI

Simultaneous Delivery of Econazole, Terbinafine and Amorolfine with Improved Cutaneous Bioavailability: A Novel Micelle-Based Antifungal “Tri-Therapy”.

Si Gou, Michel Monod, Denis Salomon, and Yogeshvar N. Kalia

Validation of HPLC-UV method

Specificity

Figure S1 presents the chromatograms obtained for blank solvent, a mixture of standard solution containing econazole (ECZ, 50 μg/mL), terbinafine (TBF, 50 μg/mL) and amorolfine (AMF, 50 μg/mL). The method was specific for (i) ECZ (RT=2.54 min), (ii) TBF (RT=4.18 min) and (iii) AMF (RT=6.54 min); quantified using detection wavelengths of 214 nm, 224 nm and 219 nm, respectively.

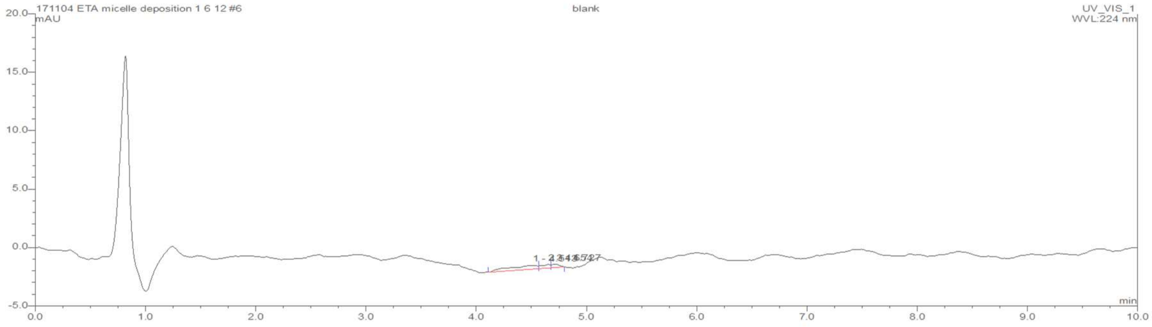

(a)

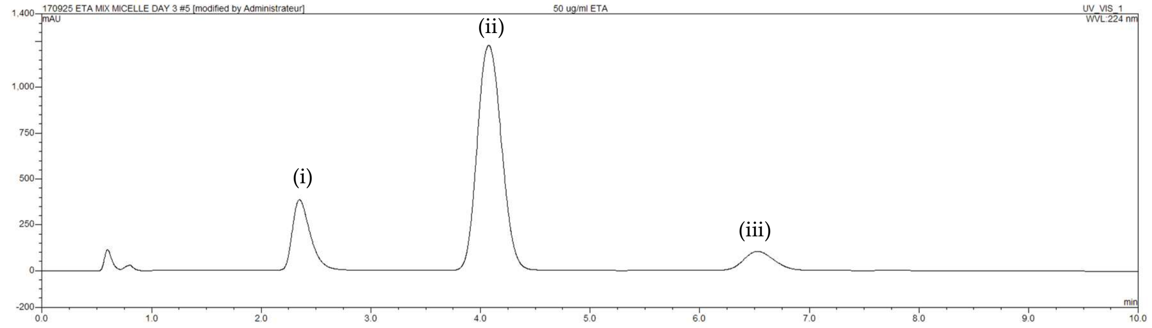

(b)

Figure S1. Chromatograms of (a) blank solvent, (b) a mixture of (i) ECZ (50 μg/mL), (ii) TBF (50 μg/mL) and (iii) AMF (50 μg/mL) standard.

Limit of detection and limit of quantification

The limit of detection (LOD) and limit of quantification (LOQ) were determined using the linear regression method and found to be 1.0 and 5.0 µg/mL for econazole, 1.0 and 5.0 µg/mL for terbinafine and1.0 and 5.0 µg/mL for amorolfine, respectively.

Linearity

Good linearity was observed in a concentration range of 10-100 µg/mL for econazole, terbinafine and amorolfine with R^2 values of of 0.9995, 0.9987, and 0.999, respectively.

Precision and accuracy

Intra- and inter-day accuracy and precision were determined using 10, 50 and 100 µg/mL standards (**Table S1**).

Table S1. Intra- and inter-day accuracy and precision for simutaneous quantification of ECZ, TBF and AMF in solvent (values are given as mean ± SD).

	**Intra-day**			**Inter-day 1**			**Inter-day 2**		
**$[ECZ]_{Theo}$ (µg/mL)**	$[ECZ]_{Meas}$ (µg/mL)	RSD (%)	Recovery (%)	$[ECZ]_{Meas}$ (µg/mL)	RSD (%)	Recovery (%)	$[ECZ]_{Meas}$ (µg/mL)	RSD (%)	Recovery (%)
10	9.87 ± 0.19	2.23	98.68	8.97 ± 5.15	2.66	89.69	9.81 ± 0.22	2.66	98.10
50	49.12 ± 0.67	1.41	100.27	46.27 ± 4.14	9.58	100.76	49.69 ± 0.30	0.62	99.38
100	100.4 ± 1.65	1.67	98.23	101.6 ± 7.45	7.55	92.53	100.2 ± 0.92	0.94	100.16
**$[TBF]_{Theo}$ (µg/mL)**	$[TBF]_{Meas}$ (µg/mL)	RSD (%)	Recovery (%)	$[TBF]_{Meas}$ (µg/mL)	RSD (%)	Recovery (%)	$[TBF]_{Meas}$ (µg/mL)	RSD (%)	Recovery (%)
10	11.47 ± 1.23	14.41	114.71	10.43 ± 0.97	13.01	104.30	8.55 ± 0.24	2.13	85.50
50	47.62 ± 1.69	3.79	97.40	47.82 ± 2.23	4.98	95.63	52.71 ± 1.98	3.57	105.43
100	101.1 ± 6.66	6.79	95.23	100.8 ± 6.45	6.59	100.87	98.80 ± 8.52	8.39	98.80
**$[AMF]_{Theo}$ (µg/mL)**	$[AMF]_{Meas}$ (µg/mL)	RSD (%)	Recovery (%)	$[AMF]_{Meas}$ (µg/mL)	RSD (%)	Recovery (%)	$[AMF]_{Meas}$ (µg/mL)	RSD (%)	Recovery (%)
10	10.60 ± 0.55	7.14	106.02	10.33 ± 0.46	6.56	103.28	9.86 ± 0.12	2.09	98.63
50	47.46 ± 0.66	1.48	94.93	49.18 ± 3.60	7.87	98.35	50.19 ± 0.30	1.60	100.82
100	101.3 ± 1.01	1.03	101.25	100.4 ± 4.43	4.57	100.35	102.2 ± 1.60	1.86	102.23

Validation of UHPLC-MS/MS method

Specificity

Figure S2 presents the chromatogram obtained for blank solvent, blank skin matrix and a mixture of standard solution containing ECZ (5 ng/mL), TBF (5 ng/mL) and AMF (5 ng/mL). No ECZ, TBF or AMF was found in the skin sample. The method was considered as specific for ECZ (381.07→125.01 transition), TBF (292.27→115.04 transition) and AMF (318.35→105.02 transition) quantification in skin samples. The three compounds were eluted at (i) TBF (RT=1. 64 min), (ii) ECZ (RT=1.88 min), (iii) AMF (RT=2.16 min) and the peaks were clearly separated from the solvent front and skin matrix.

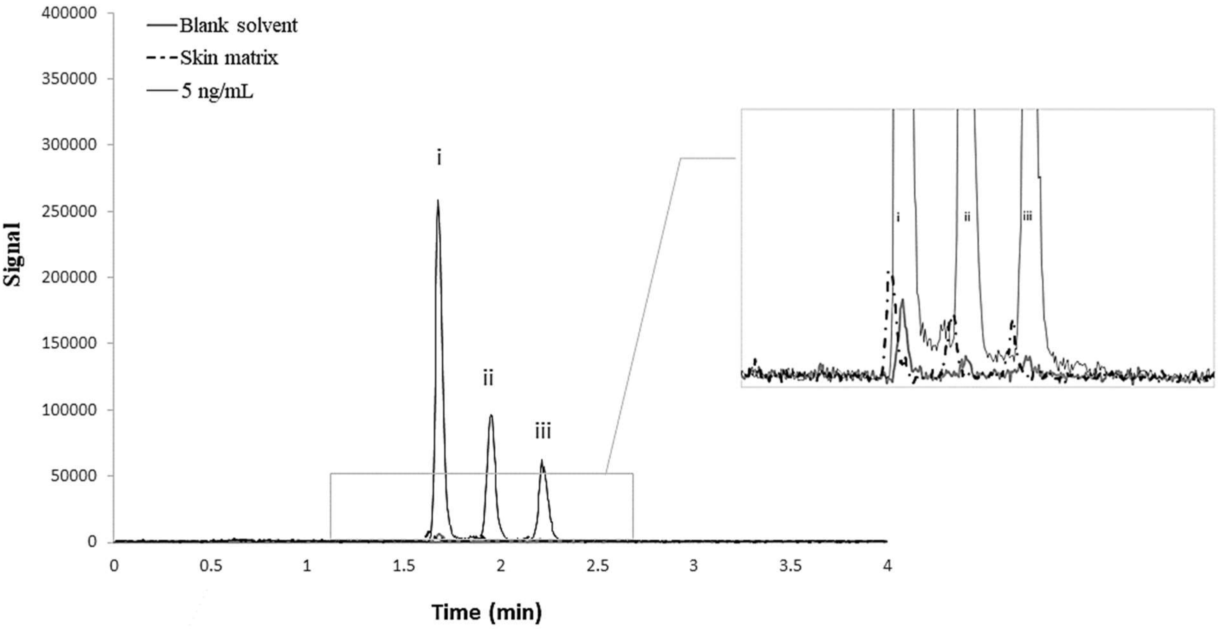

Figure S2. MRM monitoring for (i) TBF (292.27→115.04 transition), (ii) ECZ (381.07→125.01 transition), and (iii) AMF (318.35→105.02 transition) in skin sample. Chromatograms of blank solvent, skin matrix and a mixture of ECZ (5 ng/mL), TBF (5 ng/mL) and AMF (5 ng/mL) standard.

Limit of detection and limit of quantification

The limit of detection (LOD) and limit of quantification (LOQ) were determined using the linear regression method and found to be 1.0 and 2.0 ng/mL for ECZ, 1.0 and 2.0 ng/mL for TBF and 1.0 and 2.0 ng/mL for AMF, respectively.

Linearity

The method was found to be linear in the concentration range of 2.0 ~ 100 ng/mL with a R^2 of 1 for ECZ, TBF and AMF. The detection was performed using UHPLC-MS/MS and the mass spectrometer settings are provided in **Table S2**.

Table S2. MS/MS settings for detection of ECZ, TBF or AMF.

**Parameters**	**ECZ**	**TBF**	**AMF**
Nature of the parent ion	[M+H] +	[M+H] +	[M+H] +
Parent ion (m/z)	381.07	292.27	318.35
Daughter ion (m/z)	125.01	115.04	105.02
Collision Energy (V)	26	50	38
Cone voltage (V)	38	28	10
Capillary voltage (kV)	1.5	1.5	1.5
Desolvation temperature (°C)	350	350	350
Desolvation gas flow (L/h)	650	650	650
Collision gas flow (L/h)	0.15	0.15	0.15
LM resolution 1	3	3	3
HM resolution 1	15	15	15
Ion energy 1 (V)	0.4	0.4	0.4
LM resolution 2	2.93	2.93	2.93
HM resolution 2	15.13	15.13	15.13
Ion energy 2 (V)	0.8	0.8	0.8

Precision and accuracy

Intra- and inter-day accuracy and precision were determined using 5, 10 and 50 ng/mL standards (**Table S3**).

Table S3. Intra- and inter-day accuracy and precision for simutaneous quantification of ECZ, TBF and AMF in skin samples (values are given as mean ± SD).

	**Intra-day**			**Inter-day 1**			**Inter-day 2**		
**$[ECZ]_{Theo}$ (µg/mL)**	$[ECZ]_{Meas}$ (µg/mL)	RSD (%)	Recovery (%)	$[ECZ]_{Meas}$ (µg/mL)	RSD (%)	Recovery (%)	$[ECZ]_{Meas}$ (µg/mL)	RSD (%)	Recovery (%)
5	5.05 ± 0.26	5.19	100.94	4.43 ± 0.26	4.90	88.70	4.73 ± 0.14	2.59	96.46
10	10.11 ± 0.37	3.70	101.14	9.81 ± 0.49	4.66	98.12	10.08 ± 0.37	3.47	103.39
50	49.66 ± 1.05	2.12	99.32	51.41 ± 0.55	1.05	102.83	50.30 ± 0.98	1.93	101.99
**$[TBF]_{Theo}$ (µg/mL)**	$[TBF]_{Meas}$ (µg/mL)	RSD (%)	Recovery (%)	$[TBF]_{Meas}$ (µg/mL)	RSD (%)	Recovery (%)	$[TBF]_{Meas}$ (µg/mL)	RSD (%)	Recovery (%)
5	4.90 ± 0.19	4.09	98.07	4.21 ± 0.38	6.94	84.17	4.38 ± 0.13	2.38	85.64
10	10.03 ± 0.36	3.61	100.31	9.68 ± 0.44	4.04	96.78	9.78 ± 0.28	2.51	95.90
50	50.42 ± 1.08	2.13	100.84	52.08 ± 0.12	0.23	104.17	51.37 ± 1.92	3.64	100.03
**$[AMF]_{Theo}$ (µg/mL)**	$[AMF]_{Meas}$ (µg/mL)	RSD (%)	Recovery (%)	$[AMF]_{Meas}$ (µg/mL)	RSD (%)	Recovery (%)	$[AMF]_{Meas}$ (µg/mL)	RSD (%)	Recovery (%)
5	5.10 ± 0.16	3.12	101.92	4.56 ± 0.56	10.97	91.12	4.80 ± 0.16	3.14	98.26
10	10.08 ± 0.16	1.61	100.76	9.72 ± 1.00	9.74	97.21	9.94 ± 0.35	3.36	101.88
50	49.68 ± 0.82	1.64	99.36	51.35 ± 3.46	6.66	102.69	50.74 ± 1.71	3.35	103.91

Validation of antifungal solubility in the receiver compartment

The solubility of ECZ, TBF and AMF in PBS and PBS containing 0.1%, 0.5 % and 1.0 % Tween 80 was determined by the validated UHPLC-MS/MS method, results are shown in **Table S4**.

Table S4. Antifungal solubility test.

Medium	Solubility (mg/mL)		
	ECZ	TBF	AMF
PBS (0.1% Tween 80)	1.94	0.86	3.87
PBS (0.5% Tween 80)	3.65	2.91	6.97
PBS (1.0% Tween 80)	4.37	3.88	9.65

Validation of antifungal extraction from skin

Full thickness porcine skin samples (n=3; area of 0.5 cm^2) were spiked with a known amount of ECZ, TBF and AMF (0.47, 0.43, 0.48 $\mu g/cm^2$, respectively) in solution. After 2 h, skin samples were cut into small pieces and soaked in 5 mL extraction medium — ACN: Water (50:50), ACN: Water (70:30) or ACN: Water (90:10), under constant stirring at room temperature for 4 h. Skin extracts were centrifuged at 1000 rpm for 20 min, then supernatants were filtered through 0.22 µm nylon membrane and were analysed using UHPLC-MS/MS. The amount of each compound recovered are presented in Table S5.

Table S5. Recovery of ECZ, TBF and AMF from skin extraction (values are given as mean ± SD).

Extraction medium	Compound	Applied amount (µg/cm2)	Recovered amount (µg/cm2)	Recovery (%)
ACN:Water (50:50)	ECZ	0.47	0.43 ± 0.01	91.00 ± 1.87
	TBF	0.43	0.29 ± 0.05	66.90 ± 10.87
	AMF	0.48	0.33 ± 0.04	68.66 ± 8.42
ACN:Water (70:30)	ECZ	0.47	0.43 ± 0.03	90.53 ± 5.42
	TBF	0.43	0.37 ± 0.04	84.46 ± 9.48
	AMF	0.48	0.39 ± 0.04	80.86 ± 8.64
ACN:Water (90:10)	ECZ	0.47	0.46 ± 0.02	97.43 ± 3.41
	TBF	0.43	0.42 ± 0.02	96.64 ± 3.52
	AMF	0.48	0.44 ± 0.01	91.40 ± 2.18

The results indicated that the extraction medium composed of ACN:Water at a ratio 90:10 produced the best recovery profile with more than 90 % of ECZ, TBF and AMF being recovered during the extraction procedure. The extraction method was therefore considered as suitable for the extraction of the antifungal drugs in the *in vitro* skin permeation experiment.

In vitro antifungal activity against pathogenic isolates.

Table S6. *In vitro* antifungal activity of ECZ, TBF and AMF against dermatophyte, moulds and yeasts.

		ECZ (μg/mL)	TBF (μg/mL) /(mg/mL)	AMF (mg/mL)
Dermatophyte	***T. rubrum wt***	**1.31**	**0.02**	**0.08**
	T. rubrum	0.031–0.5	0.008–0.125	<0.001-0.13
	T.mentagrophytes	0.01	0.007–0.5	0.001-0.13
	T. interdigitale	0.031–0.5	0.008–0.25	
	E. floccosum	0.25	0.01–1	0.003-6.2
	T. tonsurans	0.031–0.5	0.031–0.125	
	M. canis	0.031	0.125	0.001-0.13
	M. gypseum		0.007–0.06	0.01-0.13
Yeast	*C. albicans*	100		0.001->100
	C. glabrata (*T. glabrata)*	100		0.06->100
	C. guilliermondii			0.1-2
	C. krusei	100		0.05-10
	C. parapsilosis	10		0.02-100
	C. tropicalis	100		0.001->100
	Cryptococcus neoformans	10		<0.001-8
	Pityrosporum spp. (*Malassezia spp.)*			0.005-0.5
Moulds	*Aspergillus fumigatus*		0.06–16	16->128
	A. flavus		0.03-4	30->128
	A. niger	10	0.06–2	3->100
	A. nidulans		0.12–2	3->100
	Acremonium spp.		0.06–8	0.25-2
	Fusarium spp.		0.5–16	0.3-100
	Scopulariopsis brevicaulis	1000	1-16	0.03-5
	Scytalidium spp.			0.1-1

MIC_{80} (concentration of antifungal producing a growth inhibition of 80 % or more, nmol/mL) against *T. rubrum wt* data were obtained from [1-5].

References

[1] Laurent, A. Antifungal susceptibility testing and novel pharmaco-therapeutic options for topical management of ony-chomycoses. M Pharm research project, School of Pharmaceutical Sciences, University of Geneva (2016).
[2] R.C. Heel, R.N. Brogden, T.M. Speight, G.S. Avery, Econazole: a review of its antifungal activity and therapeutic efficacy, Drugs, 16 (1978) 177-201.
[3] N. Baghi, T. Shokohi, H. Badali, K. Makimura, A. Rezaei-Matehkolaei, M. Abdollahi, M. Didehdar, I. Haghani, M. Abastabar, In vitro activity of new azoles luliconazole and lanoconazole compared with ten other antifungal drugs against clinical dermatophyte isolates, Med Mycol, 54 (2016) 757-763.
[4] G. Garcia-Effron, A. Gomez-Lopez, E. Mellado, A. Monzon, J.L. Rodriguez-Tudela, M. Cuenca-Estrella, In vitro activity of terbinafine against medically important non-dermatophyte species of filamentous fungi, J Antimicrob Chemother, 53 (2004) 1086-1089.
[5] M. Haria, H.N. Bryson, Amorolfine - a Review of Its Pharmacological Properties and Therapeutic Potential in the Treatment of Onychomycosis and Other Superficial Fungal-Infections, Drugs, 49 (1995) 103-120.
